# Hyaluronic Acid, Succinic Acid, and Exosomes in the Treatment of Facial Hyperpigmentation: A Report of Two Cases

**DOI:** 10.7759/cureus.102876

**Published:** 2026-02-03

**Authors:** Valentina Corvalan, Victor Mercado, Marta Amin

**Affiliations:** 1 Nursing, Instituto Chileno de Rejuvenecimiento y Optimización de Medicina Estética, Santiago, CHL; 2 Otolaryngology-Head and Neck Surgery, Instituto de Neurorrehabilitación y Balance, Santiago, CHL; 3 Otolaryngology-Head and Neck Surgery, Instituto Chileno de Rejuvenecimiento y Optimización de Medicina Estética, Santiago, CHL; 4 Dentistry, Instituto Chileno de Rejuvenecimiento y Optimización de Medicina Estética, Santiago, CHL

**Keywords:** hyaluronic acid, plant-derived exosomes, post-inflammatory hyperpigmentation, skin quality, succinic acid

## Abstract

Facial hyperpigmentation is a common clinical manifestation of various dermatologic conditions and may arise from multiple endogenous and exogenous factors. Its therapeutic management remains a clinical challenge due to its persistent nature and the limited long-term effectiveness of conventional treatment strategies. In recent years, emerging regenerative approaches, such as the use of exosomes and the combination of non-cross-linked hyaluronic acid with succinic acid, have been described in the international literature based on their biological, anti-inflammatory, and tissue microenvironment modulatory properties. In this case report, we present two patients with facial hyperpigmentation of similar etiology who were treated using a combined protocol based on plant-derived exosomes and non-cross-linked hyaluronic acid plus succinic acid. We describe the treatment protocol, follow-up, and the final clinical outcomes observed.

## Introduction

Cutaneous hyperpigmentation is a common dermatologic condition in which skin color becomes abnormally darkened. These changes in pigmentation may result from a wide range of endogenous and exogenous factors, including hormonal alterations, inflammation, skin injury, acne, eczema, certain medications, and ultraviolet radiation exposure [1]. This condition represents a frequent and therapeutically challenging disorder, particularly in patients with higher skin phototypes [2]. Its pathophysiology is multifactorial and involves melanocytic hyperactivity, chronic inflammation, oxidative stress, alterations of the dermal microenvironment, and extracellular matrix dysfunction, all of which contribute to disease persistence and recurrence [3].

Conventional therapies, such as topical depigmenting agents, retinoids, chemical peels, and energy-based devices, often provide partial or transient results and may be associated with skin irritation or rebound pigmentation, especially in patients with skin of color [3]. These limitations have driven the search for alternative therapeutic approaches aimed at modulating the underlying biological mechanisms involved in hyperpigmentation [3].

In this context, exosome-based therapies have emerged as a promising regenerative strategy due to their role in intercellular communication, inflammation modulation, and regulation of melanocytic activity, with reports describing sustained improvements in pigmentation and overall skin quality [4,5-10]. Complementarily, non-cross-linked hyaluronic acid combined with succinic acid has demonstrated biostimulatory and metabolic effects on dermal fibroblasts, promoting extracellular matrix remodeling, tissue homeostasis, and cutaneous repair processes [11,12-18].

Considering these mechanisms, the combined use of plant-derived exosomes and hyaluronic acid plus succinic acid may represent a biologically oriented therapeutic alternative for the management of facial hyperpigmentation [4]. Therefore, this report presents two cases of patients with post-acne facial hyperpigmentation treated using this combined therapeutic approach.

## Case presentation

Two female patients, aged 40 and 26 years, presented to our medical center in June 2025 with long-standing facial hyperpigmentation associated with acne. Both patients reported a history of inflammatory acne since adolescence, followed by persistent hyperpigmentation predominantly involving the frontal region, mid-facial area, and mandibular region. Both described a significant negative impact of these lesions on their self-esteem.

**Figure 1:**
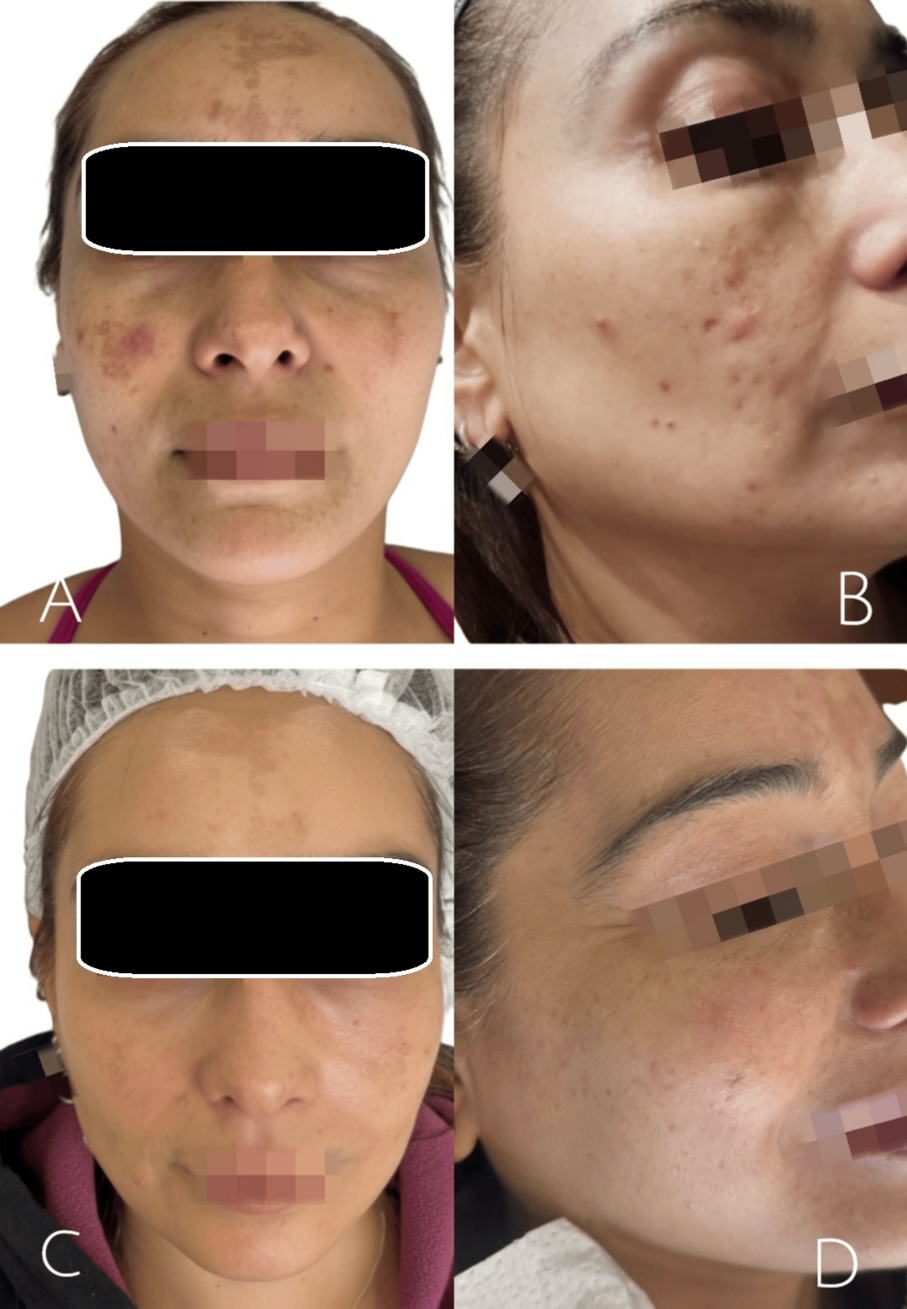
Baseline and post treatment clinical images of a patient with facial hyperpigmentation and acne related sequelae (A, B) Baseline frontal and oblique views showing facial hyperpigmentation, residual erythema, and altered skin texture. (C, D) Post-treatment frontal and oblique views demonstrate a reduction in hyperpigmentation and improved homogeneity of facial skin tone after completion of the regenerative protocol.

**Figure 2:**
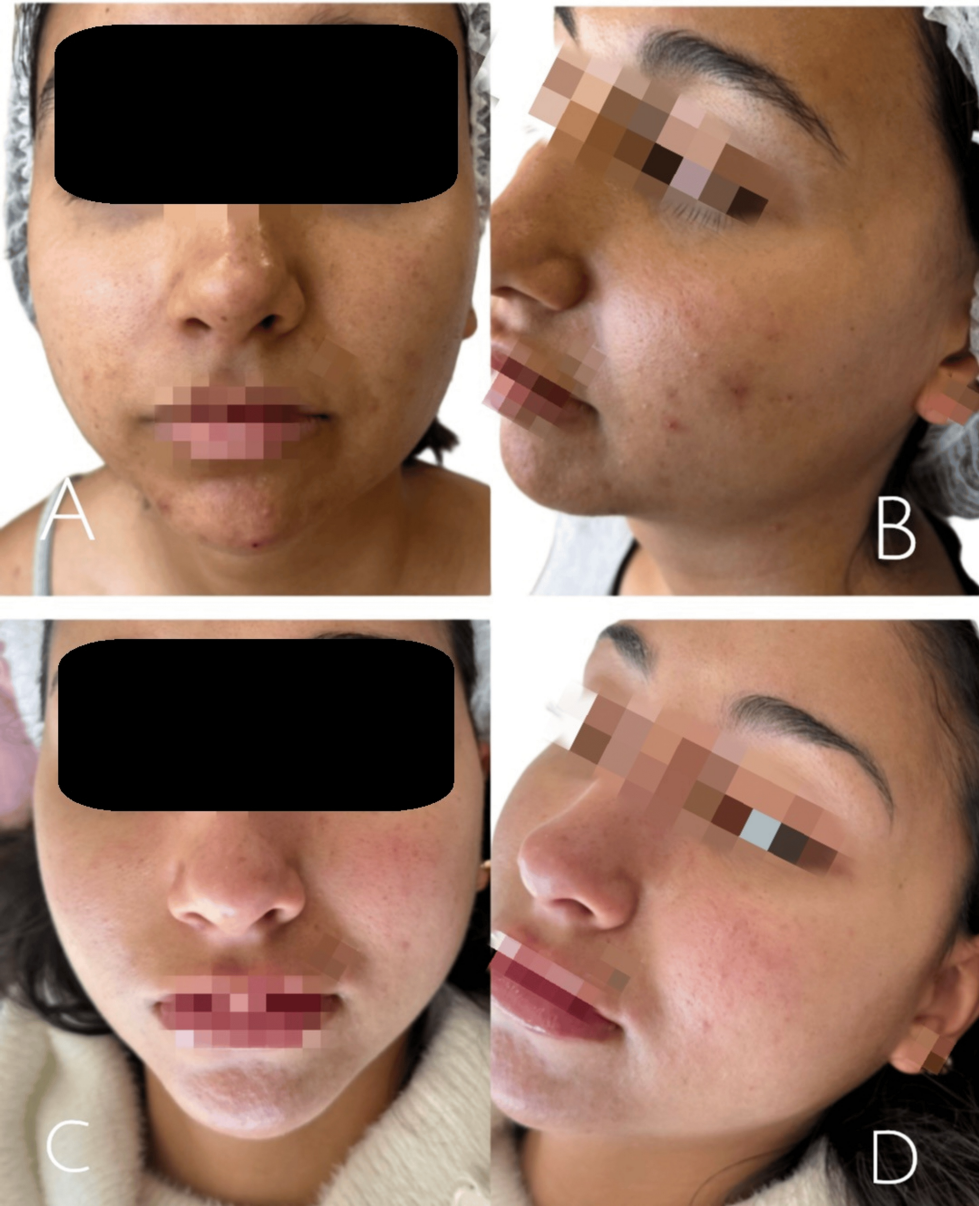
Baseline and post treatment clinical images of a patient with facial hyperpigmentation and acne related sequelae (A, B) Baseline frontal and oblique views showing facial hyperpigmentation, residual erythema, and altered skin texture. (C, D) Post-treatment frontal and oblique views demonstrate a reduction in hyperpigmentation and improved homogeneity of facial skin tone after completion of the treatment protocol.

At the initial clinical evaluation, both patients exhibited multiple hyperchromic macules, residual erythematous lesions, and altered skin texture (Figures 1A, 1B and Figures 2A, 2B). No active nodulocystic acne lesions, cutaneous infections, or relevant systemic comorbidities were identified at the time of assessment.

A standardized multimodal regenerative approach was implemented in both patients. Four treatment sessions were conducted over the same time period, with intervals of 15 days between sessions. During the first two sessions, treatment consisted of the administration of non-cross-linked hyaluronic acid combined with succinic acid (Inbiotec Amber®, IT Pharma, Chile; sanitary registration No. EDM 458/19) at a dose of 2 mL per session, delivered via intradermal microinjections using the micropapule technique. In the subsequent two sessions, plant-derived exosomes (NXO®, IT Pharma, Chile; sanitary registration No. 2601C-7/25), obtained from Panax ginseng, were applied at a dose of 3 mL per session using microneedling with a dermapen device at a depth of 0.5 mm.

Throughout the course of treatment, a progressive clinical improvement was observed in both patients. At the intermediate evaluation performed during the third session, a visible reduction in facial hyperpigmentation, attenuation of residual erythema, and improvement in skin texture and tone homogeneity were noted. At the final evaluation, conducted two and a half months after treatment initiation, both patients demonstrated marked aesthetic improvement, with smoother skin and more uniform pigmentation (Figures 1C, 1D and Figures 2C, 2D).

The severity of acne-related sequelae and post-treatment outcomes was retrospectively assessed using the Global Acne Scarring Classification system [4], based on standardized clinical photographic documentation. In Case 1, severity improved from Grade 2, corresponding to moderate severity, at baseline to Grade 1, corresponding to mild severity, after treatment. In Case 2, post-acne sequelae improved from mild to moderate severity, corresponding to Grade 1 to 2 at baseline, to mild severity, corresponding to Grade 1, following the regenerative protocol. No adverse events or treatment-related complications were recorded. Standardized clinical photographic documentation was obtained at baseline, after completion of treatment, and throughout the clinical follow-up period.

## Discussion

The pathophysiological mechanisms underlying post-inflammatory hyperpigmentation and its complex clinical behavior have been widely described in the literature. Nautiyal and Wairkar [19] reviewed the embryologic origin of melanocytes from the neural crest and their central role in the pigmentary response to cutaneous inflammation. The authors highlight that topical depigmenting agents represent first-line therapy, followed by chemical exfoliation as second-line treatment, while physical therapies, including laser-based procedures and microneedling, are generally reserved as third-line strategies due to their adverse effect profile and variable tolerability, particularly in certain skin phototypes.

In the specific context of acne-associated hyperpigmentation, Taylor et al. (2023) proposed the term acne-induced macular hyperpigmentation, emphasizing that inflammation remains active throughout the pathogenic process [20]. Through a modified Delphi consensus, the authors recommend early and effective acne treatment as a fundamental pillar, using topical retinoids and benzoyl peroxide, in addition to targeted therapies for acne induced macular hyperpigmentation such as hydroquinone, azelaic acid, chemical exfoliation, and antioxidants [20].

Similarly, Adebusoye and Srivastava extensively analyzed combination therapies for acne and post inflammatory hyperpigmentation, reporting that optimal outcomes are achieved through multimodal approaches integrating topical and systemic treatments along with procedural interventions [21]. Nevertheless, the authors underscore limitations related to treatment adherence, the occurrence of adverse effects, and variable long-term efficacy in prolonged clinical settings.

Collectively, these reviews suggest that isolated depigmenting therapies and certain physical procedures may be insufficient or poorly tolerated, particularly in patients with intermediate to high skin phototypes. This reinforces the need to explore therapeutic strategies aimed at modulating the cutaneous microenvironment and promoting underlying tissue repair rather than focusing exclusively on pigment suppression [1-3].

In parallel, cellular metabolism and persistent subclinical inflammation have gained increasing relevance in the pathophysiology of pigmentary sequelae. Recent studies have demonstrated that succinate, traditionally regarded as an intermediate of the tricarboxylic acid cycle, functions as a key mediator in inflammatory responses. Disruption of mitochondrial metabolism leads to intracellular succinate accumulation, which can stabilize hypoxia inducible factor one alpha and modulate the production of pro-inflammatory cytokines. In addition, succinate may act extracellularly as a signaling molecule through its specific receptor, succinate receptor one, regulating immune cell activation, particularly macrophage polarization toward the M2 phenotype, which is associated with anti-inflammatory and tissue reparative functions [22].

Within this framework, modulation of cellular metabolism through succinic acid is proposed as a strategy with potential impact on persistent inflammation and altered tissue environments, beyond a purely depigmenting effect. Concurrently, non-cross-linked hyaluronic acid has demonstrated biostimulatory effects on dermal fibroblasts, promoting hydration, extracellular matrix synthesis, and cutaneous reparative responses, particularly when combined with metabolically active molecules such as succinic acid [15-17].

Complementarily, exosome-based therapies have emerged as regenerative tools capable of modulating inflammation, promoting cellular proliferation, and facilitating tissue remodeling through complex intercellular signaling pathways. These mechanisms may contribute to improvements in residual pigmentation as well as overall skin quality in the context of chronic cutaneous damage [5-8].

## Conclusions

In conclusion, this case series suggests that a combined regenerative protocol using plant-derived exosomes and non-cross- linked hyaluronic acid plus succinic acid may be a feasible and well-tolerated option for the management of dermal hyperpigmentation and post-acne sequelae in selected patients. While the clinical outcomes observed were favorable, these findings should be interpreted cautiously, given the limited number of cases and the descriptive nature of the study. Further controlled studies with larger cohorts and standardized assessment tools are necessary to better define the efficacy, reproducibility, and long-term role of this combined approach in pigmentary and post acne conditions.
